# Recurrence and retreatment of anterior communicating artery aneurysms after endovascular treatment: a retrospective study

**DOI:** 10.1186/s12883-020-01871-5

**Published:** 2020-07-29

**Authors:** Chang Ki Jang, Joonho Chung, Jae Whan Lee, Seung Kon Huh, Nak-Hoon Son, Keun Young Park

**Affiliations:** 1 Department of Neurosurgery, Severance Stroke Center, Severance Hospital, Yonsei University College of Medicine, 50 Yonsei-ro, Seodaemun-gu, Seoul, 03722 Republic of Korea; 2 Severance Institute for Vascular and Metabolic Research, Yonsei University College of Medicine, Seoul, Republic of Korea; 3 Department of Neurosurgery, Muhas Academic Medical Center, Dar es Salaam, United Republic of Tanzania; 4 Medical Research Supporting Section, Yongin Severance Hospital, Yonsei University College of Medicine, Yongin, Gyeonggi-do Republic of Korea

**Keywords:** Aneurysm, Anterior communicating artery, Recurrence

## Abstract

**Background:**

**S**urgical treatment of anterior communicating artery (Acom) aneurysm is challenging due to anatomic complexity. We aimed to describe our experiences with endovascular treatment (EVT) of Acom aneurysms, and to evaluate the incidence and risk factors of recurrence and retreatment.

**Methods:**

The study comprised 260 patients who were treated at a single center between January 2010 and December 2018. Patients who had EVT, including stent-assisted coiling of Acom aneurysms, were included. All medical records were retrospectively reviewed. The incidence and risk factors of recurrence and retreatment were evaluated. Univariate and multivariate analysis were conducted.

**Results:**

Recurrence of Acom aneurysms occurred in 38 (14.6%) patients. Mean follow-up duration was 27 months (range 1–110). Multivariate logistic regression indicated that ruptured aneurysm (odds ratio [OR] 3.55, *P* = 0.001), dome direction (anterior) (OR 3.86, *P* = 0.002), maximal diameter (OR 1.19, *P* = 0.02), and mean age (OR 0.96, *P* = 0.02) were independent risk factors for aneurysm recurrence. Of 38 cases of recurrence, 10 (3.8%) patients underwent retreatment. Ruptured aneurysm (OR 14.7, *P* = 0.004), maximal diameter (OR 1.56, *P* = 0.02), inflow angle (OR 1.04, *P* = 0.03), and Raymond-Roy classes II and III (OR 6.19, *P* = 0.03) showed significant relation to retreatment in multivariate logistic regression analysis.

**Conclusions:**

In our study, recurrence rate of Acom aneurysms after EVT was 14.6%. Rupture, anterior dome direction, maximal diameter, and mean age were significantly associated with recurrence. Retreatment rate of recurrent Acom aneurysms after EVT was 3.8%. Patients with Acom aneurysms with large inflow, rupture, large size, or incomplete occlusion may be at a high risk of retreatment of recurring aneurysm.

## Background

Anterior communicating artery (Acom) aneurysm has a higher risk of rupture than the other aneurysms [1]. Therefore, treatment of Acom aneurysm is essential and critical for prevention of aneurysmal subarachnoid hemorrhage. However, surgical treatment of Acom aneurysm is still challenging due to anatomic complexity. Acom aneurysm shows a variety of anomalies and complex anatomy like fenestrated, triplicated, and azygous anterior cerebral artery. It also has many perforator and associated vessels such as the recurrent artery of Heubner, anterior lenticulostriate, and bilateral A1 and A2 arteries [2–4]. Furthermore, surgical clipping of Acom aneurysm sometimes require a dissection of the rectus gyrus or olfactory nerve.

Since endovascular treatment has emerged as a feasible and acceptable option for aneurysm treatment, Alshekhlee et al. reported that hospital mortality was higher in patients who had surgical clipping than in those who had endovascular coiling (1.6% versus 0.57%) [5]. However, retreatment after initial treatment was performed in 17.4% of patients in the endovascular treatment (EVT) group and in 3.8% of patients in the surgical clipping group in an International Subarachnoid Aneurysm Trial (ISAT) study [6]. The study also found that young age, large lumen size, and incomplete occlusion were risk factors for retreatment after EVT. O’Neil et al. reported that endovascular coiling resulted in significantly lower treatment-related morbidity compared with clipping, but clipping resulted in significantly lower angiographic recurrence and retreatment [7].

The purpose of this study was to evaluate recurrence and retreatment rates and their risk factors after endovascular treatment of Acom aneurysms.

## Methods

### Patient population

This study was approved by our Institutional Review Board, and the requirement for patients’ informed consent was waived due to its retrospective design.

Between January 2010 and December 2018, 514 Acom aneurysms were consecutively treated by either surgical or endovascular method in our institute. Patients who had vasculitis or infectious fusiform and blood blister-like aneurysms were excluded from this study. Patients who underwent a flow diverter treatment were also excluded. Finally, 260 patients with Acom aneurysms who underwent coiling with or without stents were included. All clinical and radiological data were obtained from electronic medical records and a prospectively registered aneurysm database and were retrospectively reviewed.

### Radiologic evaluation

At the initial coiling, all aneurysms were evaluated with 3D angiogram (Allura Xper FD20/20 and Allura Clarity, Philips Medical Systems, Best, The Netherlands). Based on 3D angiogram findings, aneurysm factors including maximal size, size ratio, inflow angle, hypoplastic A1, and dome direction were evaluated. Size ratio was calculated by dividing the aneurysm maximal size (mm) by the average size of both A1 segments (mm) [8]. Inflow angle was defined as the angle between the maximal height of the aneurysm and the parent vessel [9]. Hypoplastic A1 was defined as A1 with diameter less than 50% of the diameter of contralateral A1 [10]. Additionally, we dichotomized aneurysm direction into anterior or posterior by drawing an imaginary perpendicular line to the anterior cranial fossa using sagittal view of computed tomography (CT) angiography [11]. All radiologic images were retrospectively reviewed by two investigators with consensus. For the correction of different values between the two investigators, mean values were obtained.

After the initial coiling, routine imaging follow-up was conducted with 3.0-T magnetic resonance angiography (MRA) at 6, 18, 30, and 60 months. Depending on each patient’s clinical situation, further follow-up was allowed as well as post-treatment and follow-up angiographic occlusion grade were assessed according to Raymond-Roy classification, wherein class I is defined as complete occlusion, class II as neck remnant, and class III as sac remnant [12]. Recurrence was defined as any progression of Raymond-Roy class or increasing of aneurysmal flow. All class III aneurysms on MRA were further evaluated with 3D angiogram.

### Retreatment

Retreatment was considered for aneurysms if volume of recurred sac was ≥20.0 mm^3^ on 3D angiogram. Retreatment modality was planned and decided based on consensus by a multidisciplinary team.

### Statistical analysis

Univariate analysis was performed to determine the association of any recurrence with other factors. The statistical significance of recurrence was analyzed using the chi-square test for categorical, nominal variables, or the logistic regression test for continuous, numerical variables. Multivariate logistic regression analysis was performed for variables with an unadjusted effect and a *P*-value < 0.20 in univariate analysis to determine independent associations of recurrence and retreatment with other factors. Results of binary logistic regression were reported as odd ratios (ORs) with *P*-value < 0.05 for a 95% confidence interval (CI), which was considered statistically significant. Additionally, correlation analysis and chi-square test were performed to identify the strength of relationships between age and other risk factors. All statistical analyses were performed with SPSS version 19.0 (IBM Corp., Armonk, NY, USA).

## Results

A total of 260 patients (male: female = 134:126; mean age, 57.6 years) with Acom aneurysms underwent coiling procedure. Among them, 157 (60.3%) patients had hypertension, and 115 (44.2%) were smokers. There were 183 (70.4%) unruptured and 77 (29.6%) ruptured aneurysms. Overall, mean maximal diameter of aneurysms was 5.3 mm (range 2–16.6), and mean size ratio was 2.41 (range 0.9–8.5). Mean inflow angle was 148.6° (range 75–180), and hypoplastic A1 was noted in 82 (31.5%) patients.

All aneurysms were successfully treated without (*n* = 208, 80.0%) or with (*n* = 52, 20.0%) stents, and immediate post-procedural angiogram showed 167 (64.3%) Raymond-Roy class I, 75 (28.8%) class II, and 18 (6.9%) class III aneurysms.

### Recurrence

Follow-up MRA or 3D angiogram was performed for all patients (mean 27 months; range 1–110). Recurrence was noted in 38 (14.6%) patients. In univariate analysis, age (OR 0.96, 95% CI 0.93 to 0.99, *P* = 0.01), rupture status (OR 3.66, 95% CI 1.81 to 7.52, *P* = 0.001), maximal diameter (OR 1.20, 95% CI 1.06 to 1.37, *P* = 0.005), size ratio (OR 1.50, 95% CI 1.17 to 1.95, *P* = 0.001), and dome direction (anterior) (OR 2.94, 95% CI 1.38 to 6.86, *P* = 0.007) were significant risk factors for recurrence. In multivariate analysis, age (OR 0.96, 95% CI 0.93 to 0.99, *P* = 0.02), rupture status (OR 3.55, 95% CI 1.62 to 7.91, *P* = 0.001), maximal diameter (OR 1.19, 95% CI 1.03 to 1.39, *P* = 0.02), and dome direction (anterior) (OR 3.86, 95% CI 1.67 to 9.94, *P* = 0.002) were statistically significant. These results are shown in Table 1.

**Table 1:** Risk factors for recurrence after endovascular coiling of anterior communicating artery aneurysm

				Univariate analysis		Multivariate analysis	
(*N* = 260)		Recurrence (*n* = 38)	No recurrence (*n* = 222)	OR (95% CI)	*P*-value	OR (95% CI)	*P*-value
Patient factor	Male	24 (63.1%)	110 (49.5%)	1.75 (0.87–3.63)	0.12		
	Age (year)	53.58 ± 11.7	58.3 ± 10.84	0.96 (0.93–0.99)	0.01*	0.96 (0.93–0.99)	0.02*
	Hypertension (%)	25 (65.7)	132 (59.4)	1.31 (0.65–2.77)	0.46		
	Smoking (%)	15 (39.4)	100 (45.0)	0.80 (0.39–1.59)	0.52		
Aneurysm factor	Rupture (%)	21 (55.2)	56 (25.2)	3.66 (1.81–7.52)	0.001*	3.55 (1.62–7.91)	0.001*
	Maximal diameter	6.3 ± 3.0	5.17 ± 2.1	1.20 (1.06–1.37)	0.005*	1.19 (1.03–1.39)	0.02 *
	Size ratio	3.0 ± 1.6	2.3 ± 1.0	1.50 (1.17–1.95)	0.001*	1.07 (0.69–1.68)	0.76
	Inflow angle	152.8 ± 23.0	147.9 ± 29.1	1.01 (0.99–1.02)	0.11		
	Dome direction (Anterior, %)	29 (76.3)	116 (52.5)	2.94 (1.38–6.86)	0.007*	3.86 (1.67–9.94)	0.002*
	Hypoplastic A1 (%)	13 (34.2)	69 (31.0)	1.15 (0.54–2.35)	0.70		
Treatment factor	Stent usage (%)	5 (13.1)	47 (21.1)	0.56 (0.19–1.41)	0.25		
	Raymond class (II or III, %)	18 (47.3)	75 (33.7)	1.76 (0.87–3.54)	0.10		

*OR* Odds ratio, *CI* Confidence interval

* Variable significantly related to recurrence

Correlation analysis and chi-square test performed for evaluation of any relation between age and other risk factors. As a result, *P*-value of correlation analysis between age and maximal diameter was 0.068, which was not statistically significant at the significance level of 5%, but there was a positive trend between age and maximal diameter. The correlation of other factors of ruptured status and dome direction with age were also not statistically significant.

### Retreatment

Among the 38 recurrent cases, 10 (3.8%) showed a recurred volume ≥ 20.0 mm^3^ that needed a retreatment. Mean interval between initial coiling and recurrence ≥20.0 mm^3^ was 24 ± 18 months (range 2–63 months). Retreatment was performed at 1–6 months after recurrence. One patient underwent clipping surgery. Three patients were treated with EVT using double catheter technique (Case 1), and the other six patients with EVT using stent assisted coiling (SAC) (Case 2). In univariate analysis, age, ruptured status, maximal diameter, and Raymond-Roy class II or III were statistically significant. In multivariate analysis, ruptured status (OR 14.7, 95% CI 2.77 to 127.9, *P* = 0.004), maximal diameter (OR 1.56, 95% CI 1.05 to 2.36, *P* = 0.02), inflow angle (OR 1.04, 95% CI 1.01 to 1.09, *P* = 0.03), and Raymond-Roy class II or III (OR 6.19, 95% CI 1.19 to 40.8, *P* = 0.03) were significantly associated with retreatment. These are shown in Table 2.

**Table 2:** Risk factors for retreatment after endovascular coiling of anterior communicating artery aneurysm

				Univariate analysis		Multivariate analysis	
(N = 260)		Retreatment (n = 10)	No retreatment (*n* = 250)	OR (95% CI)	*P*-value	OR (95% CI)	*P*-value
Patient factor	Male	4 (40%)	130 (52%)	0.62 (0.15–2.21)	0.46		
	Age (year)	50.7 ± 12.3	57.9 ± 10.9	0.94 (0.89–1.00)	0.04*	0.95 (0.88–1.01)	0.12
	Hypertension (%)	6 (60)	151 (60.4)	0.98 (0.27–3.93)	0.97		
	Smoking (%)	4 (40)	111 (44.4)	0.83 (0.21–2.99)	0.78		
Aneurysm factor	Ruptured (%)	8 (80)	69 (27.6)	10.4 (2.55–70.6)	0.003*	14.7 (2.77–127.9)	0.004*
	Maximal diameter	7.15 ± 3.7	5.27 ± 2.2	1.25 (1.02–1.50)	0.01*	1.56 (1.05–2.36)	0.02 *
	Size ratio	2.9 ± 2.0	2.3 ± 1.1	1.35 (0.87–1.90)	0.11		
	Inflow angle	162.8 ± 27.8	148.0 ± 28.8	1.02 (1.00–1.06)	0.12	1.04 (1.01–1.09)	0.03*
	Dome direction (Anterior, %)	7 (70)	138 (55.2)	1.89 (1.51–8.94)	0.036	5.03 (0.97–36.6)	0.07
	Hypoplastic A1 (%)	3 (30)	79 (31.6)	0.93 (0.20–3.43)	0.91		
Treatment factor	Stent usage (5)	1 (10)	51 (20.4)	0.43 (0.02–2.39)	0.43		
	Raymond class (II or III, %))	6 (60)	87 (34.8)	2.81 (1.78–11.24)	0.01*	6.19 (1.19–40.8)	0.03*

*OR* Odds ratio, *CI* Confidence interval

* Variable significantly related to retreatment

### Case presentation

#### Case 1

**Fig. 1:**
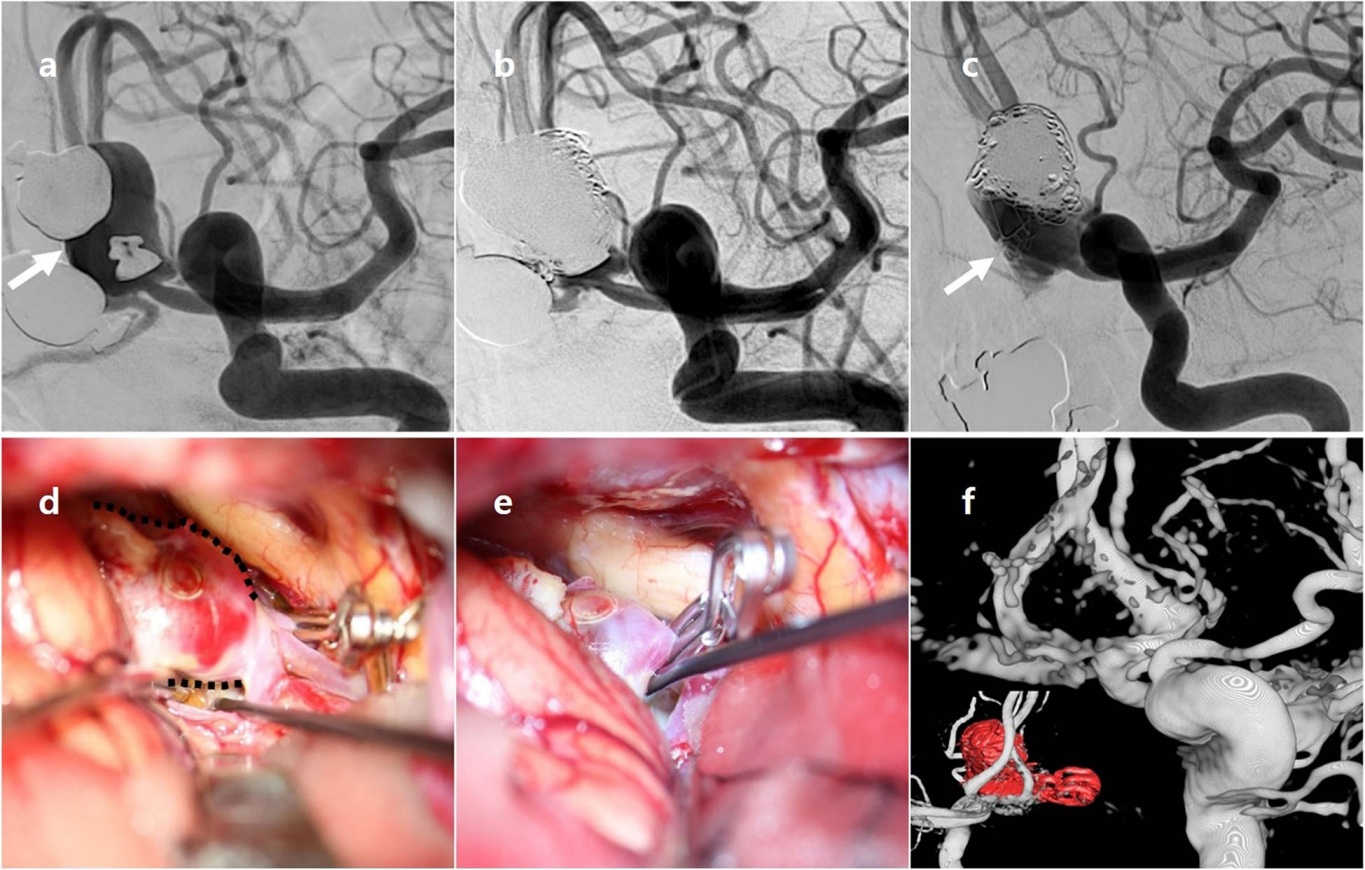
A 55-year-old patient with recurrent Acom aneurysm. Initial angiogram (**a**) showed a ruptured Acom aneurysm (arrow); maximal height 16.6 mm, neck size 6.6 mm. Immediate post-embolization image (**b**) showed a neck remnant (class II) occlusion of aneurysm without distal sac flow. After one year, cerebral angiography (**c**) revealed a recurrence of aneurysm (arrow, class III). Intraoperative image (**d**) showed recurrent aneurysm sac (dotted line), and temporary clipping was applied for exploration of aneurysm sac. Permanent clipping was done, and there was no remnant sac (**e**). On post-operative 3D reconstructive image (**f**), no remnant sac was noted. Acom = anterior communicating artery

A 55-year-old female patient underwent an emergency CT scan due to mental change. Ruptured Acom aneurysm was noted. Maximal size of aneurysm was 16.6 mm and neck size 6.6 mm. Emergent EVT was performed and immediate post-embolization angiogram showed neck remnant occlusion (class II). Patient recovered well and she was discharged without neurological deficit. After 1 year, cerebral angiography revealed a recurrence of aneurysm (class III). Surgical clipping was done without neck remnant, and both A2 segments were safely preserved. No more recurrence was seen after 2 years. These findings are described in Fig. 1.

#### Case 2

**Fig. 2:**
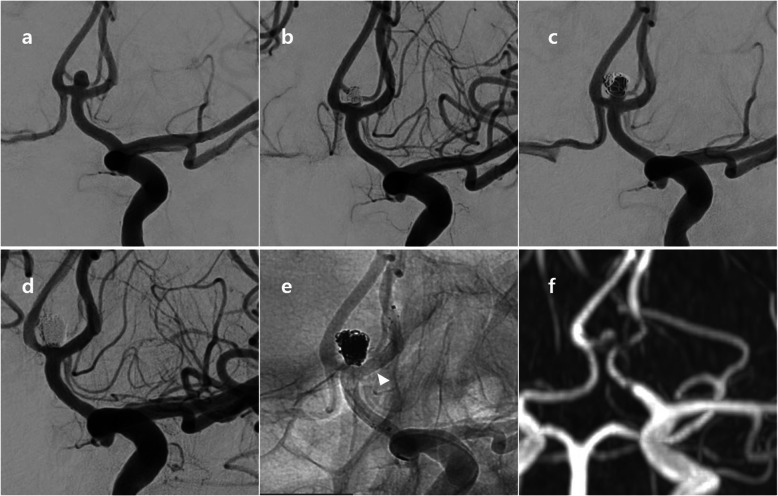
A 57-year-old patient with recurrent Acom aneurysm. Initial angiogram showed a ruptured Acom aneurysm; maximal height 4.5 mm, neck size 3.1 mm (**a**). Immediate post-embolization image (**b**) showed neck remnant occlusion without sac filling (class II). After one year, cerebral angiography (**c**) revealed a recurrence of aneurysm. Stent-assisted coiling (arrowhead) was successfully performed, but neck remnant was still observed (**d** and **e**). After one year after retreatment, MRA revealed a more recurrence of aneurysm neck. Acom = anterior communicating artery; MRA = magnetic resonance angiography

A 57-year-old male patient came into the emergency room with severe bursting headache. Ruptured Acom aneurysm was noted. Maximal size of aneurysm was 4.5 mm and neck size was 3.1 mm. Emergent EVT was performed and immediate post-embolization angiogram showed neck remnant occlusion (class II). After 1 year, cerebral angiography revealed a recurrence of aneurysm. Ipsilateral A1–2 stent-assisted coil embolization was done with neck remnant occlusion of sac (class II). After 1 year after retreatment, MRA revealed a more recurrence of aneurysm neck. These findings are described in Fig. 2.

## Discussion

This study evaluated the incidence and risk factors of recurrence and retreatment of anterior communicating artery (Acom) aneurysm after endovascular treatment. We found that the recurrence rate of Acom aneurysm after EVT was 14.6%, and its risk factors were younger age, rupture status, aneurysm size, and anterior dome direction. The rate of retreatment was 3.8% and large inflow angle, ruptured status, aneurysm size, and incomplete aneurysm occlusion were risk factors of retreatment.

Some studies have shown that surgical procedure for Acom aneurysm can lead to post-operative deficits such as memory impairment and personality changes [13, 14]. Ramos et al. also reported that gyrus rectus resection could cause cognitive and psychiatric dysfunction caused by orbital prefrontal cortex lesion or a disconnection in the ventromedial circuits [15]. Another key problem of surgery of Acom aneurysm is an olfactory nerve injury. In some cases of Acom aneurysms, especially superior or posterior direction, the frontal base is retracted to some degree in order to access the aneurysm. During the frontal lobe retraction, the olfactory nerve can be easily detached from the cribriform plate. Park et al. reported an objective olfactory dysfunction rate of 10.8% after Acom aneurysm surgery [16]. Considering the risks of these complications, minimal invasive surgery like keyhole approach with eyebrow or palpebral incision was introduced in Acom aneurysm surgery [17–19]. These approaches minimized a brain retraction and rectus gyrectomy. Some authors even advocated for use of orbitotomy approach, [20–22] and recently, endoscopic endonasal approach was reported for Acom aneurysm surgery [23].

However, surgical clipping of Acom aneurysm is still challenging, because vascular variations such as multiple fenestrated or azygous artery are common in Acom complex [24–27]. The prevalence of duplication of Acom was 18% and that of fenestration of Acom was 12–21% [28]. Surgical clipping of Acom is difficult when variations are present [2]. Thus, endovascular treatment emerged as an alternative option for Acom aneurysm treatment.

O’Neill et al. reported that coiling was significantly related to lower rate of treatment-related morbidity compared with clipping (0.8% in coiling versus 4.4% in clipping; *P* = 0.001), whereas clipping was significantly related to lower angiographic recurrence (4.9% in coiling versus 0% in clipping; *P* = 0.001) in systematic analysis [7]. Many other studies also reported that EVT was associated with a higher rate of recurrence and retreatment than clipping [29–31].

### Recurrence

Large aneurysm, rupture status, incomplete occlusion, posterior circulation, and branch artery incorporation are well-known risk factors for aneurysm recurrence after EVT [12, 32]. Park et al. reported that total recurrence and retreatment rates were 25.7% (44/171) and 10.5% (18/171), respectively after EVT of saccular aneurysm larger than 8 mm [33]. They revealed that large size, rupture status, low dome-to-neck ratio, and initial incomplete occlusion status were independent risk factors for recurrence. In the present study, age, rupture status, large aneurysm size, and anterior dome direction were significant risk factors for recurrence.

Corns et al. reported that younger age predisposed to a higher risk of recurrence in ruptured aneurysm [34]. However, their study did not give definite explanation for this observation. In some studies, younger age was a predictor of growth of aneurysm after clipping, [35, 36] but this age-related growth is not well-known. Our study also showed that younger patients had more recurrence than older patients. This age-related growing or recurrence of aneurysm can be explained in two ways. First, there was a bias toward more frequent surveillance imaging in younger patients. Second, there is a possibility that age can be correlated with other factors that signify recurrence. Therefore, we performed a correlation analysis between age and the other risk factors, and none of risk factors correlated with age significantly.

In the present study, anterior dome direction was a risk factor of Acom aneurysm recurrence. Anterior dome direction is known to reflect aneurysm hemodynamics, including wall shear stress and flow velocity, which play important roles in the growth and rupture of aneurysm [37]. This may explain its significant association with recurrence in this study.

Smoking is known as one of the most important risk factors for formation and rupture of intracranial aneurysm, [38–40] and that association was explained by inhibitory effect of cigarette smoke on alpha 1-antitrypsin [41]. However, Brinjikji et al. reported that smoking was not an independent risk factor for aneurysm recurrence (OR = 1.04, *P* = 0.87) and retreatment (OR = 0.82, *P* = 0.50) for patients receiving EVT for aneurysm [42]. On the contrary, Futchko et al. found that history of smoking—whether current or former—was associated with a significantly increased risk of aneurysm recurrence. In the study of Futchko et al., the odds ratios for aneurysm recurrence in current and former smokers were 2.73 and 2.69, respectively, compared with non-smokers. The authors accounted for the difference between the Brinjikji et al’s study and Futchko et al’s study, in that the former study exclusively used balloon-assisted coiling, whereas the later study used stent-assisted coiling [43]. Our study could not reveal any association between smoking and aneurysm recurrence.

Using computational fluid dynamic (CFD) analysis, Merih et al. identified inflow angle as an independent and robust rupture status differentiator in intracranial aneurysm [9]. CFD showed that increasing inflow angle led to deeper migration of flow with higher peak flow velocities and a greater transmission of kinetic energy into the dome. Wenjun et al. also revealed that an inflow angle of over 90° and incomplete occlusion were associated with aneurysm recurrence in unruptured aneurysm after EVT. Our study could not reveal that inflow angle was related with recurrence. However, inflow angle was associated with retreatment (*P* = 0.03). Similarly, incomplete occlusion (Raymond class II or III) was associated with retreatment (*P* = 0.03) but not with recurrence. In the present study, it seems that the relatively small number of retreatment events (*n* = 10) had the most impact on the result. Thus, in our opinion, both inflow angle and incomplete occlusion seem to have little association with recurrence, but are eventually associated with retreatment.

### Limitation

This study had several limitations. First, the decision to retreat could rely on the clinician’s decision, and it may lead to bias. However, to minimize subjective decision and bias, retreatment was considered for aneurysms with a recurred volume ≥ 20.0 mm^3^ on 3D angiogram. Furthermore, retreatment was decided based on consensus by a multidisciplinary team. Second, recurrence and retreatment cases were few compared with the other group, which showed no recurrence and retreatment. This could be a factor in lowering the statistical reliability of the results. Third, the inflow angle might be inaccurate, even though we used 3D angiogram. For the correction of this inaccuracy, mean values were applied in this study. Finally, inflow angle could change after stent placement. However, in this study, only 52 patients (20%) were treated with SAC and this could not affect the results.

## Conclusions

In our study, recurrence rate of Acom aneurysm after endovascular treatment was 14.6%. Younger age, rupture status, aneurysm size, and anterior dome direction were significantly associated with recurrence. Retreatment rate of Acom aneurysm after EVT was 3.8%. Large inflow angle, ruptured status, aneurysm size, and incomplete aneurysm occlusion were risk factors of retreatment. These patients need to be closely followed up after recoiling.

## Data Availability

The datasets used and analyzed during the current study are available from the corresponding author on reasonable request. (kypark78.md@gmail.com) Public access to the database is open on reasonable request.

## Abbreviations

Acom: Anterior communicating artery aneurysm
CFD: Computational fluid dynamic
CI: Confidence interval
CT: Computed tomography
EVT: Endovascular treatment
ISAT: International Subarachnoid Aneurysm Trial
MRA: Magnetic resonance imaging
OR: Odd ratio
SAC: Stent assisted coiling
